# Supplementary material for: A Hydrolase Produced by Rhodococcus erythropolis HQ Is Responsible for the Detoxification of Zearalenone
Source: Toxins (Basel). 2023 Dec 7;15(12):688. doi: 10.3390/toxins15120688 (PMC10747462; doi:10.3390/toxins15120688)
Supplement: Supplementary file 1 [file toxins-15-00688-s001.zip › toxins-2736058-supplementary.pdf]

toxins

MDPI

Supplementary material: A hydrolase produced by *Rhodococcus erythropolis* HQ is responsible for the detoxification of zearalenone

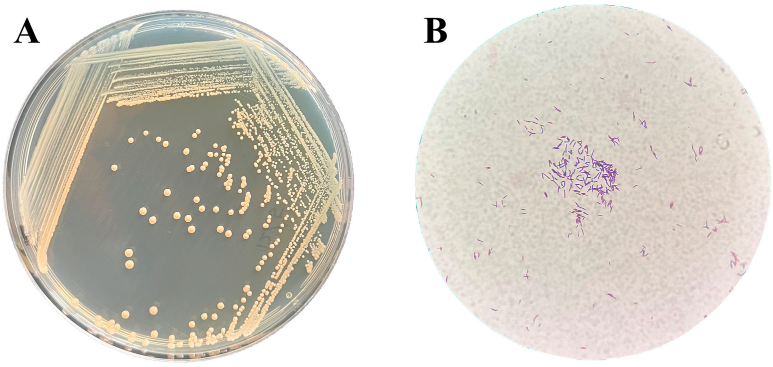

Figure S1. The morphological identification (**A**) and gram stain observation (×1000, **B**) of strain HQ. The colony morphology of strain HQ was cultured on LB plate at 30°C for three days. Strain HQ belonged to gram-positive bacteria with a rod-shaped morphology.

.

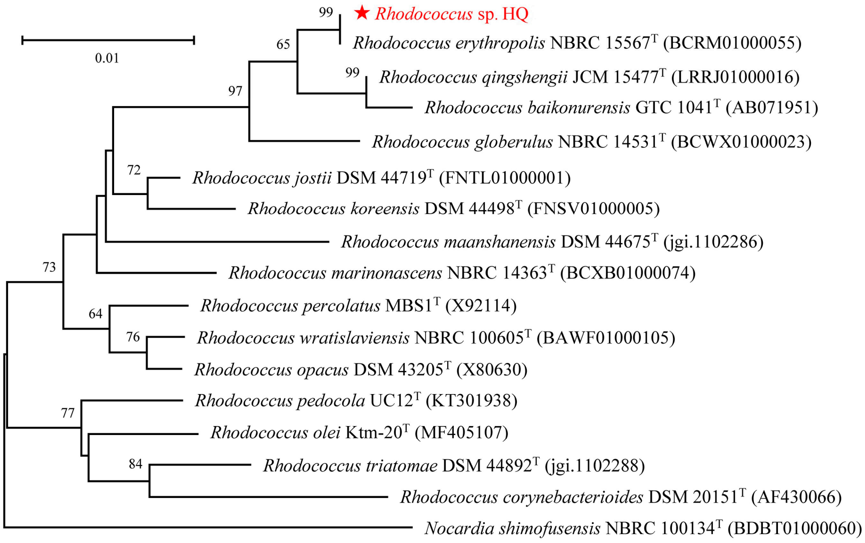

Figure S2. Phylogenetic tree of strain HQ based on 16S rRNA gene sequences, showing the relationship of strain HQ to related taxa. GenBank registrations are given in parentheses. Percent bootstrap values (1000 replicates) greater than 50% are displayed on nodes.
The red five-pointed star represents the target strain and T represents the standard strain.

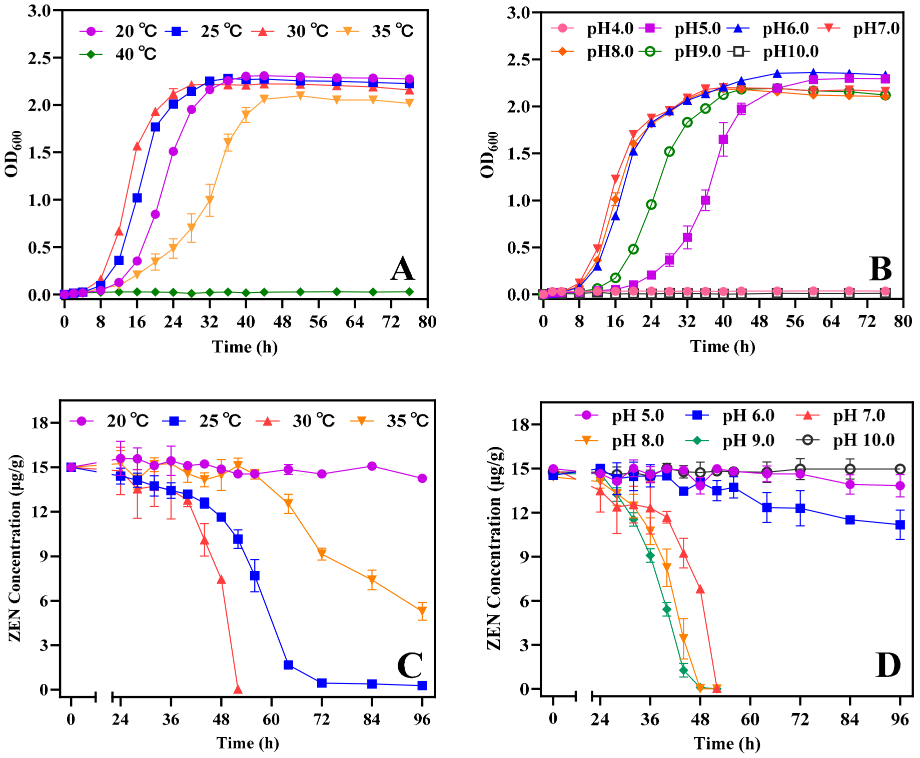

Figure S3. The growth and degradation characteristics of strain HQ in different conditions. Growth curves of strain HQ at various temperatures (**A**) and pH levels (**B**), where LB medium was used to cultivate strain HQ. ZEN degradation curves of strain HQ at various temperatures (**C**) and pH levels (**D**), where the minimal salt medium containing 15 μg/mL ZEN was used to determine the degradation efficiency of strain HQ.

.

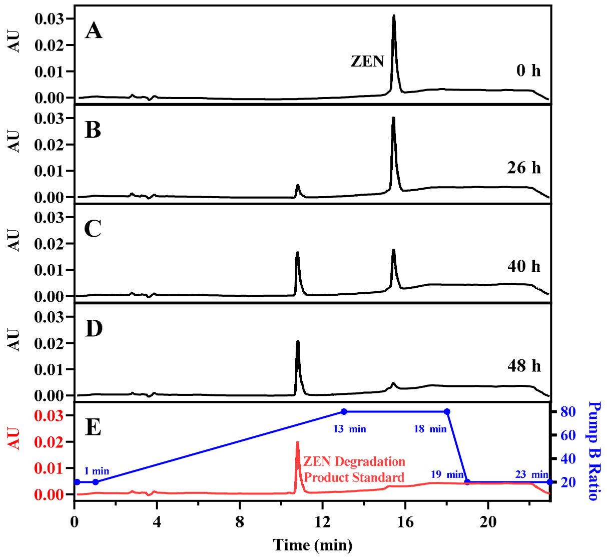

Figure S4. HPLC analysis of ZEN and its degradation product (P1) for various reaction times. (**A**) ZEN standard; (**B-D**) ZEN was degraded by strain HQ for 26 h, 40 h, and 48 h, respectively. (**E**) ZEN degradation product standard isolated by preparative HPLC from ZEN degradation by ZenH.

The HPLC conditions are as follows: separations were performed on an Eclipse XDB-C18 column (ODS, 4.6 mm × 250 mm, Zorbax). Eluent A was water, B was acetonitrile, and both eluents contained 0.05% trifluoroacetic acid. The flow rate was 0.8 mL/min, and the detection wavelength was 236 nm. The elution procedure is shown in the blue line in Figure S4E, with the initial proportion of eluent B being 20%, which was linearly increased to 80% and held for 5 min, and then returned to the initial proportion.

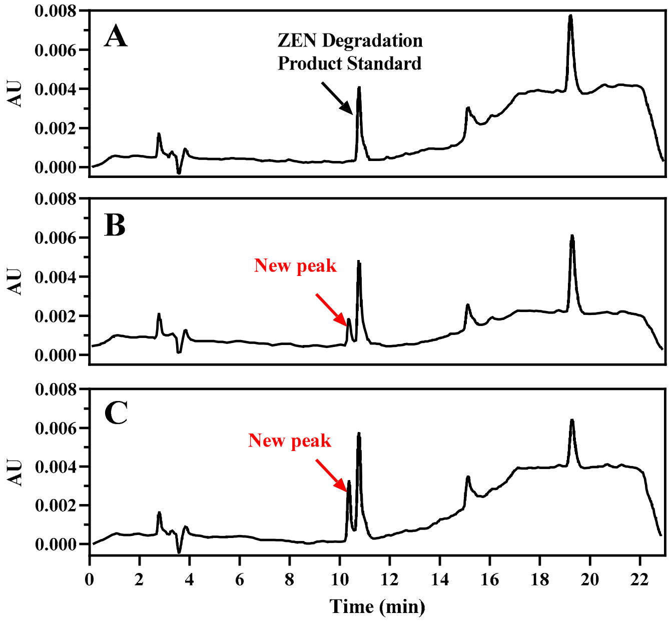

Figure S5. HPLC analysis of ZEN degradation product (P1) and its by-product (P2). Figure **A, B** and **C** correspond to the degradation products of ZEN stored in acetonitrile, water, and methanol, respectively.

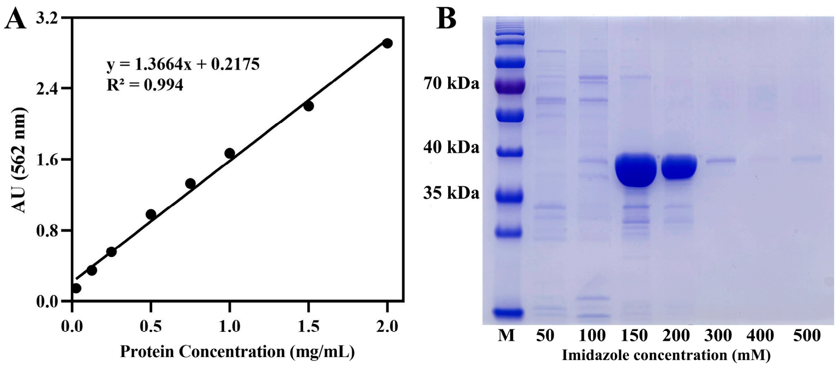

Figure S6. Protein standard curve (**A**) and SDS-PAGE of ZenR eluted at various imidazole concentrations (**B**).

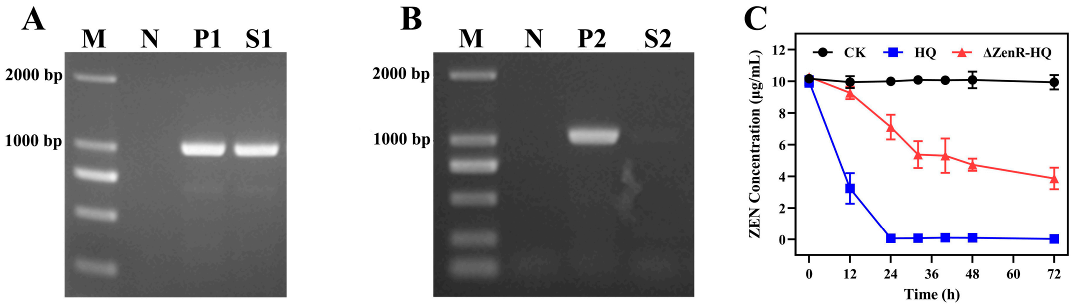

Figure S7. PCR results for the knockdown of zenR in strain HQ (**A & B**) and the ZEN degradation ability of strain HQ and its mutant ΔzenR-HQ (**C**). Figure 3427. Q); lane N is the negative control with water as the PCR template; lane P1 is the positive control with plasmid pEX18Gm-ΔzenR as the PCR template; lane S1 is the tested sample with HQ1-pEX18Gm-ΔzenR genomic DNA as the PCR template, and all of the above lanes were used to amplify the SacB gene using the SacB-F/R primer pair. Figure B: lane M is a DNA marker (TaKaRa Co., Ltd., catalog No. 3427Q); lane N is the negative control with water as the PCR template; lane P2 is the positive control with genomic DNA of strain HQ1 as the PCR template; lane S2 is the tested sample with ΔzenR-HQ1 genomic DNA as the PCR template, and all of the above lanes were used to amplify the zenR gene using the zenR-F/R primer pair.

.

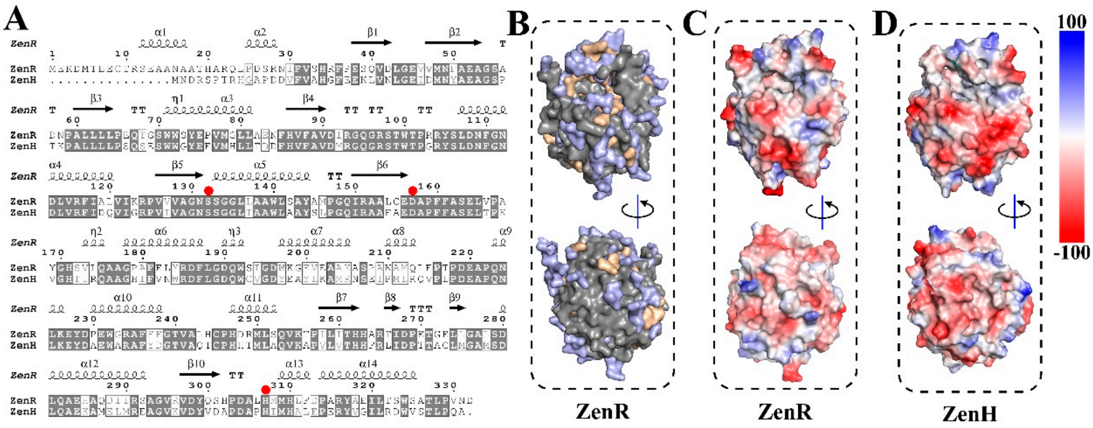

Figure S8. Sequence alignment of ZenR and ZenH (A), ZenR protein surface visualization model based on the alignment of ZenR and ZenH amino acid sequences (B), and electrostatic potential diagrams of ZenR and ZenH protein surfaces (C & D). A, catalytic triads of ZenH are denoted by red circles; B, the Light purple amino acid residues in the model are the amino acid residues that are inconsistent in the two sequences, the wheat-colored are the similar amino acid residues, and the grey is the amino acid residues that are consistent; C&D, electrostatic potential diagrams of enzymes, where red represents the negative electrostatic potential (negatively charged), while blue represents the positive electrostatic potential (positively charged). All three models (B, C, and D) displayed both the front and rear of the enzyme.

.

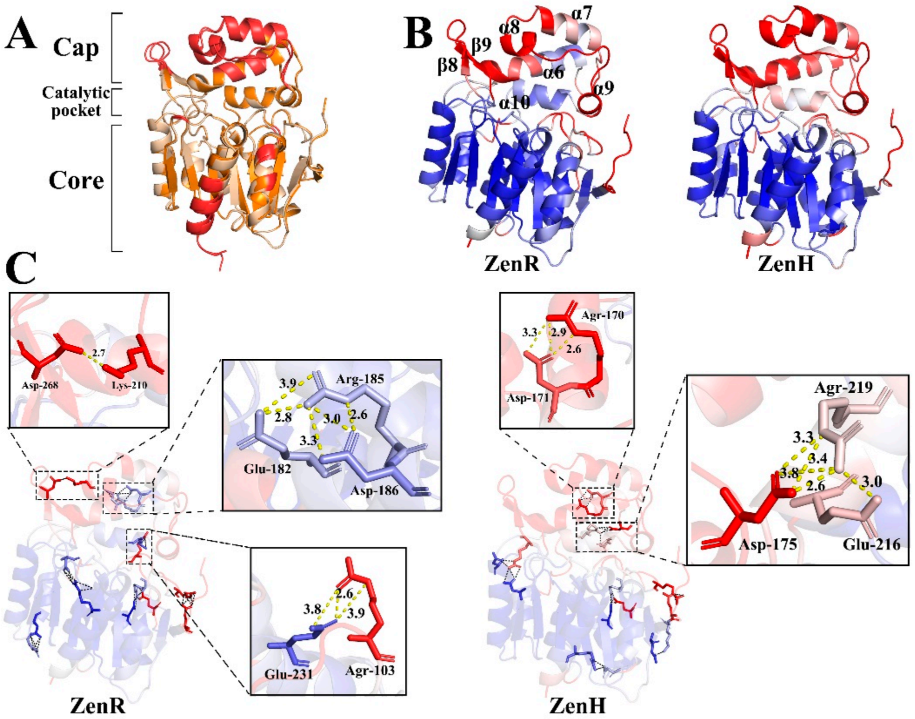

Figure S9. Diagrams superimposing the structures of the two enzymes (**A**). Diagram of the enzyme structure based on B-factor coloring (**B**), and an illustration of the salt bridge on the enzyme surface (**C**). A, the color of ZenR is wheat, the color of ZenH is orange, and the non-overlapping structures are marked in red; B, the red portion in the enzyme structure represents the high B-factor, whereas the blue portion represents the low B-factor; C, the image in the black box depicts the salt bridge generated by the interaction of amino acid residues on the enzyme surface, and the yellow line represents the length of the salt bridge.

.

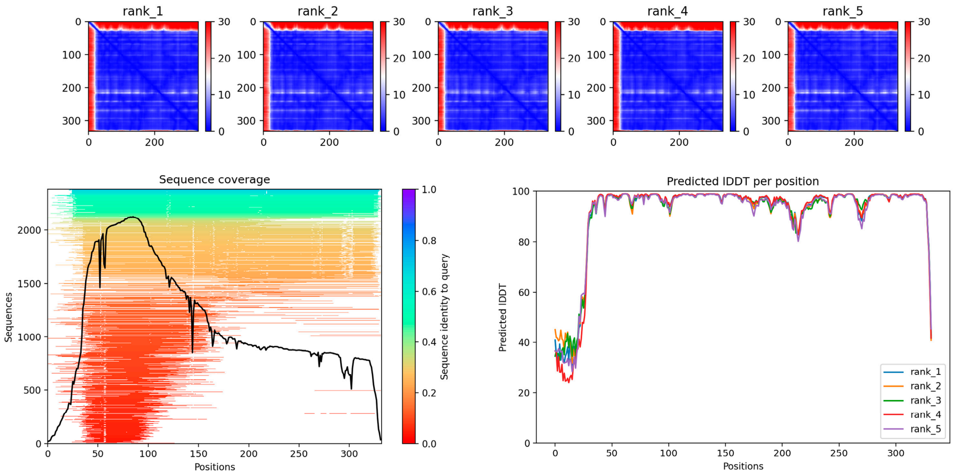

Figure S10. The evaluation of the ZenR predicted structural model. A, Coverage of ZenH amino acid sequences by the template; B, Score with pLDDT of each amino acid site in the prediction model (Regions with pLDDT value below 50 are considered to be low quality); and C, Prediction alignment error (PAE) for each amino acid in the prediction model;. Model I has a score of 93.1; Model IV and V have a score of 93.5; Model II has a score of 93.7 and Model III has a score of 93.9.

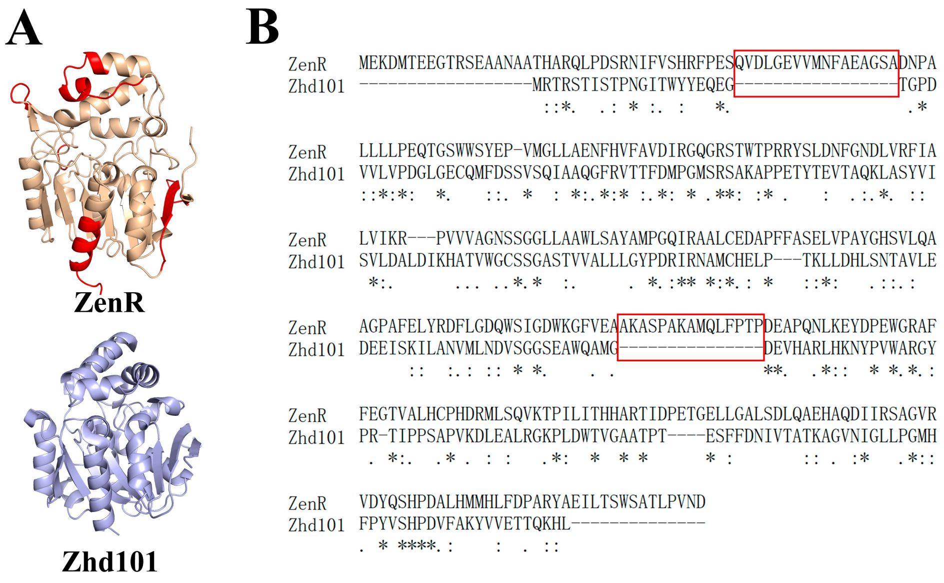

Figure S11. Structural models of ZenR and Zhd101 (A), and sequence alignment of ZenR and Zhd101 (B). A, the color of ZenR is wheat, the color of Zhd101 is light purple, and the red portion of the ZenR structure corresponds to the additional amino acid sequence identified in ZenR compared to Zhd101. B, the sequence highlighted by red box represents the additional amino acid sequence in comparison to Zhd101.

.

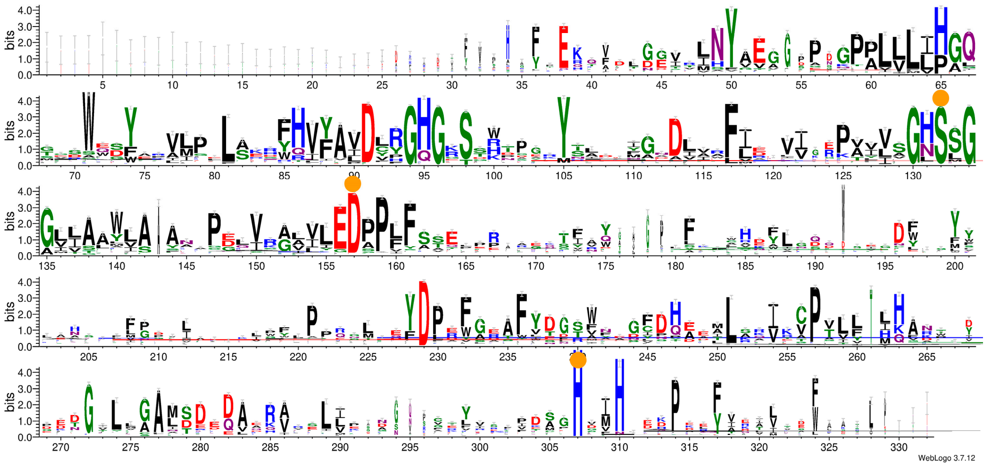

Figure S12. Predictions on the conservation of amino acids in the ZenR protein. Orange circles designate catalytic triads. The graphic was generated using the Consensus Finder Web and Weblogo 3.0.

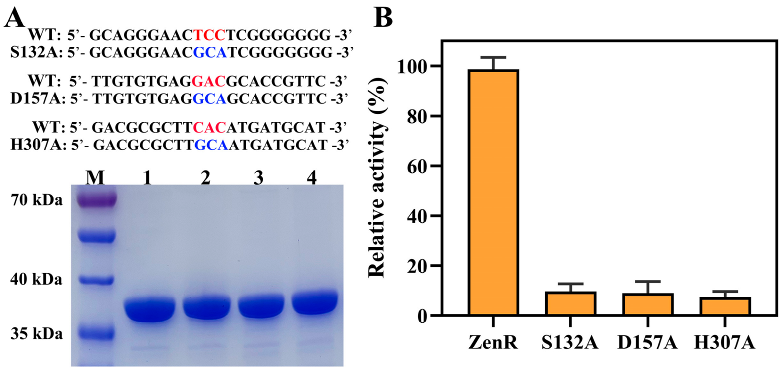

Figure S13. SDS-PAGE analysis of ZenR and its mutants (**A**), functional verification of ZEN degradation of ZenR and its mutants (**B**). lane M is a protein marker (Thermo Fisher Scientific, catalog No. 26616); Lane 1: BL21-pET29a-*zenR*; Lanes 2-4: BL21-pET29a-zenH mutants S132A, D157A and H307N, respectively.

Table S1. Biochemical characteristics of strain HQ analyzed by VITEK 2 compact bacterial identification system.

Test items	Result	Test items	Result
Ala-Phe-Pro-arylamidase	-	D-Galactose	-
Ornithine decarboxylase	+	Arginine GP	+
Phenylalanine arylamidase	+	Pyruvate	+
Beta-Galactosidase	-	D-Glucose	-
L-Pyrrolydonyl-arylamidase	-	D-Maltose	+
Tyrosine arylamidase	-	D-Mannitol	-
Beta-Glucosidase	+	D-Melezitose	-
Beta-Xylosidase	+	Citrate (sodium)	-
L-Proline arylamidase	+	5-Bromo-4-chloro-3-indoxyl-beta-glucuronide	-
Lipase	-	D-Sorbitol	-
Alpha-Mannosidase	-	L-Malate	+
Urease	+	D-Ribose	-
Alpha-Glucosidase	+	Maltotriose	-
Alpha-Galactosidase	-	L-Glutamine	+
Glycine arylamidase	-	Phenylphosphonate	-
Beta-D-Fucosidase	-	Coumarate	-
Esculin hydrolyse	-	2-keto-D-Gluconate	-
Succinate alkalinisation	+	D-Xylose	-
L-Lactate alkalinisation	+	Sucrose	-
O/129 Resistance (comp. vibrio.)	-	D-Trehalose	-
Ellman	-		

Note: "+" indicates a positive result; "-" indicates a negative result. According to the analysis, strain HQ is *Rhodococcus erythropolis*.

Table S2. Strains and plasmids used in this study.

Strain, plasmid	Description	Source or reference
**Strain**		
Rhodococcus sp. HQ	Wild type	From soil
HQ-pEX18Gm-ΔzenR	Strain HQ integrated the suicide plasmid pEX18Gm-ΔzenR	This study
ΔzenR-HQ	Strain HQ with knockout of the *zenR* gene	This study
Escherichia coli BL21(DE3)	F^- *ompT hsdS* (r_B^- m_B^-) *gal dcm lacY1* (DE3)	Sangon Biotech Co., Ltd.
E. coli DH5α	F^- *recA1 endA1 thi-1 supE44 relA1 deoR Δ(lacZYA-argF)U169 Φ 80dlacZ*ΔM15	Sangon Biotech Co., Ltd.
E. coli BL21(DE3)-*zenR*	*E. coli* BL21(DE3) harboring the plasmid pET29a-*zenR*	This study
Bacillus subtilis WB800	*trpC2 nprE aprE epr bpr mpr nprB*	Preserved in lab
WB800-pAX01-*nprE zenR*	*Bacillus subtilis* WB800 integrated the plasmid pAX01-*nprE zenR*	This study
**Plasmids**		
pEX18Gm	pEX18GM is a SacB suicide plasmid, Gm^r	Preserved in lab
pEX18Gm-ΔzenR	pEX18Gm derivative carrying the upstream and downstream regions of *zenR*, Gm^r	This study
pET29a (+)	Expression vector, Km^r	Preserved in lab
pET29a-*zenR*	pET29a (+) derivative carrying *zenR*, Km^r	This study
pET29a-S132A	pET29a (+) derivative carrying *zenR*-S132A, Km^r	This study
pET29a-D157A	pET29a (+) derivative carrying *zenR*-D157A, Km^r	This study
pET29a-H307A	pET29a (+) derivative carrying *zenR*-H307A, Km^r	This study
pAX01	pAX01 is a shuttle plasmid for protein expression from *Bacillus* species, Em^r	Preserved in lab
pAX01-*nprE* zenR	pAX01 derivative carrying signal peptide gene *nprE* and zenR, Em^r	This study

Km^r, kanamycin resistant; Gm^r, Gentamicin resistant; Em^r, Erythromycin resistance

Table S3. Primers used in this study.

Primer	Sequence (5′–3′)	Purpose
27F	AGAGTTTGATCCTGGCTCAG	To amplify the 16S ribosomal RNA gene
1492R	TACGGCTACCTTGTTACGACTT	
zenR-F	AACTTTAAGAAGGAGATATAATGGAGAAGGACATGACCG	Amplify *zenR* gene with pET29A homology arms
zenR-R	TGCTCGAGTGCGGCCGCGTCGTTCACAGGCAGTGT	
LF-F	AATTCGAGCTCGGTACCCGGCCTACGAGGGGAGTCCAACC	Amplify the upstream region of *zenR*
LF-R	GACAGTTCCTGGTTGCGGTTGGGTTGCTATCAAG	
RF-F	ACCCAACCGCAACCAGGAACTGTCCCCACTAAC	Amplify the downstream region of *zenR*
RF-R	TAAAACGACGGCCAGTGCCCAGTCAACGGCGCCATCAATAT	
SacB-F	GAACCAAAAGCCATATAAGGAAAC	Amplify *SacB* gene with
SacB-R	CGTCAATCGTCATTTTTGATCC	
nprE-F	AAATCAAAGGGGGAAATGGTGGGTTTAGGTAAGAAATTGTCTG	Amplify *nprE* gene
nprE-R	GGTCATGTCCTTCTCCATAGCCTGAACACCTGGCAG	
zenR-Bac-F	ATGGAGAAGGACATGACCG	Amplify *zenR* gene
zenR-Bac-R	GGCCGCCCGCGGGAGCTCCTAGTCGTTCACAGGCAGTGTTG	
S132A-F	GCATCGGGGGGGCTGCTGG	Amplify mutant gene *zenR*-S132A with Ser132 replaced Ala132
S132A-R	GTTCCCTGCCACGACGACAGG	
D157A-F	GCAGCACCGTTCTTTGCGTCGGAG	Amplify mutant gene *zenR*-D157A with Asp157 replaced Ala157
D157A-R	CTCACACAATGCTGCACGGATCTGG	
H307A-F	GCAATGATGCATCTGTTCGACCCCGCTC	Amplify mutant gene *zenR*-H307A with His307 replaced Ala 292
H307A-R	AAGCGCGTCGGGGTGC	
